# Erythem, Petechien, Juckreiz und Schwellung im Genitalbereich

**DOI:** 10.1007/s00105-024-05300-3

**Published:** 2024-02-05

**Authors:** Alena Ropele, Peter Wolf, Daisy Kopera

**Affiliations:** Klinik für Dermatologie, Medizinische Universität Graz, Auenbruggerplatz 8, 8036 Graz, Österreich

## Anamnese

Die 32-jährige Patientin stellte sich aufgrund von Erythemen mit Juckreiz und Schwellung der Handflächen, der Fußsohlen, des Genitalbereiches sowie des Gesäßes vor. Die Symptomatik bestand zum Zeitpunkt der Vorstellung seit 3 Tagen, und der Juckreiz hatte 10 min nach einem (allergologisch unauffälligen) Mittagessen begonnen. Aufgrund des Verdachts auf eine anaphylaktische Reaktion war die Patientin initial im nächstgelegenen Krankenhaus vorstellig geworden und hatte dort parenteral ein Antihistaminikum und Glukokortikosteroide erhalten. Die Symptomatik hatte sich nur gering gebessert. In der Folge stellte sich die Patientin in der dermatologischen Ambulanz zusätzlich mit Fieber, Gelenk‑/Muskelschmerzen, Müdigkeit, Mattigkeit und Abgeschlagenheit sowie starken brennenden Schmerzen genital vor. Die Anamnese ergab keine bekannten Allergien, keine Vorerkrankungen, keine Dauermedikation und keine Einnahme von Bedarfsmedikation innerhalb der letzten 14 Tage. Die Drogenanamnese war negativ.

## Klinischer Befund

**Abb. 1:**
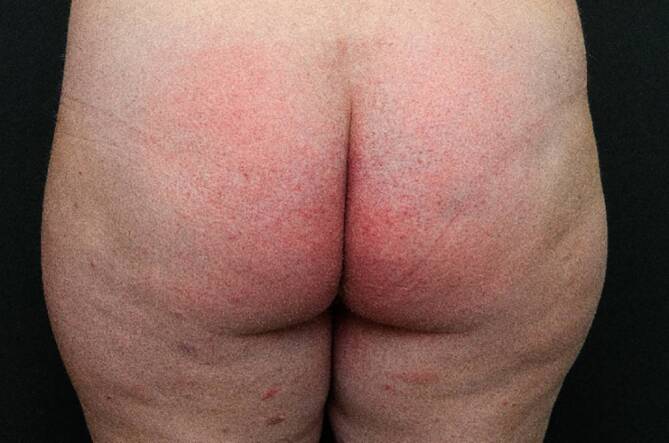
Erythematöses Exanthem gluteal mit petechialer Komponente

**Abb. 2:**
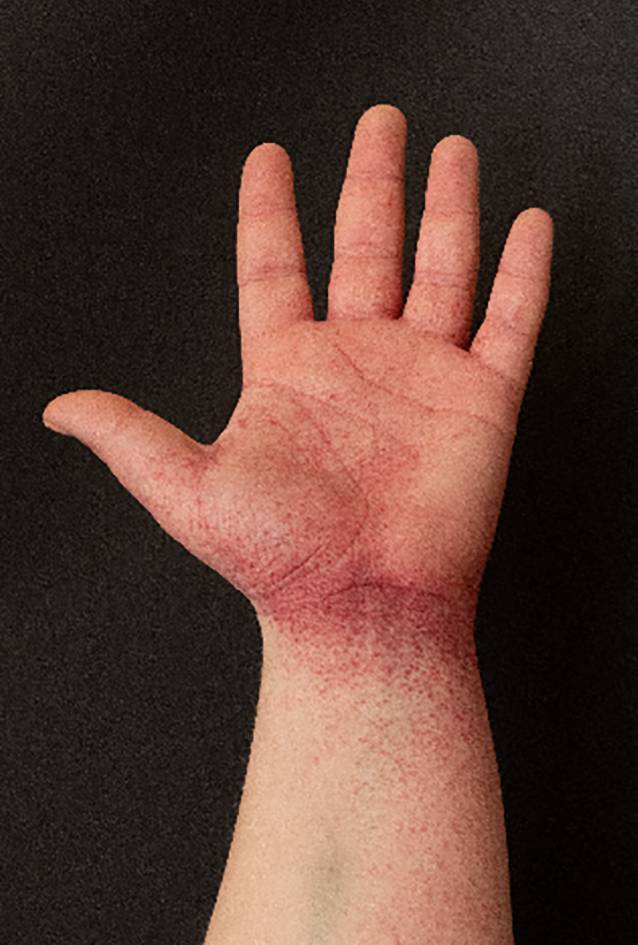
Handflächen angeschwollen mit einem vom Handgelenk auslaufenden petechialen Erythem

Bei der klinischen Untersuchung zeigte sich in den Intertrigines und am Gesäß (Abb. 1) sowie an den Handflächen übergreifend auf den Unterarm und an den Fußsohlen ein erythematöses Exanthem mit teilweise petechialer Komponente (Abb. 2 und 3). Im Gesicht fanden sich ein Wangenerythem mit Gesichtsschwellung und Stippchen an der Mundschleimhaut. Eine Lippen- oder Zungenschwellung war nicht vorhanden, und die Uvula war schmal und mittig. Es lag kein Globusgefühl oder eine subjektive Dyspnoe vor. Die Temperatur betrug 39,7 °C.

**Abb. 3:**
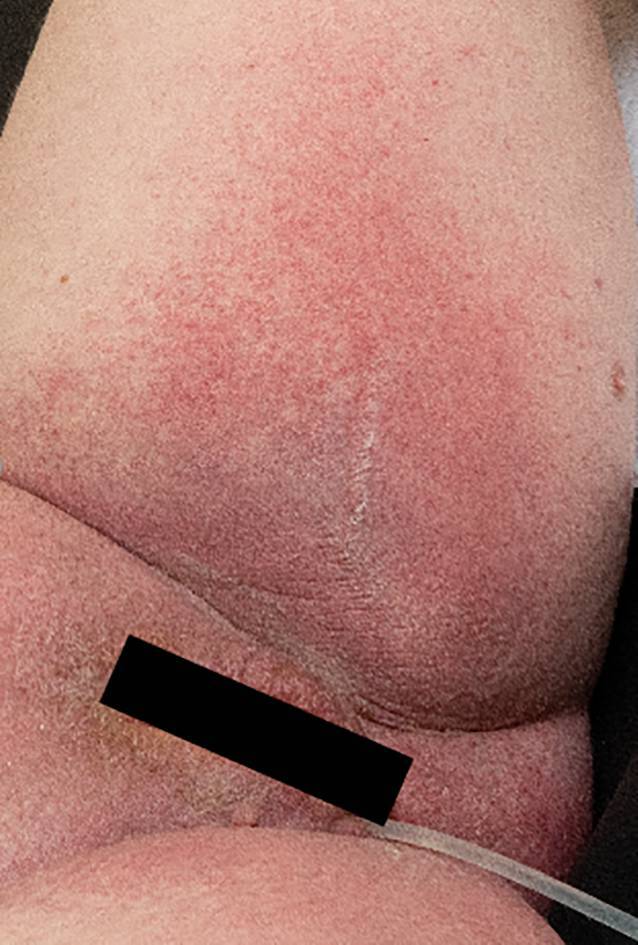
Genitalbereich deutlich angeschwollen und gerötet

## Labor

- Leukozyten 5,73 G/l, neutrophile Granulozyten 78 % (mäßig erhöht, Normalwert: 50–75 %), Lymphozyten 7 % (vermindert, Normalwert: 20–40 %), CRP (C-reaktives Protein) 39,0 mg/l (+, mäßig erhöht: Normalwert: < 5,0)
- Parvovirus B19 EIA(„enzyme immunoassay“)-Ig(Immunglobulin)M und EIA-IgG negativ; PCR (Polymerasekettenreaktion) Parvovirus > 6,1E + 10 cop/ml (++++ positiv)

## Wie lautet Ihre Diagnose?

## Weiteres Prozedere

Die Patientin wurde aufgrund des schlechten Allgemeinzustandes und einer zunehmenden Oxygenierungsstörung stationär aufgenommen. Eine durchgeführte Thoraxröntgenaufnahme zeigte eine Lobärpneumonie.

**Diagnose: **Infektionsinduziertes beugenbetontes, an Baboon-Syndrom erinnerndes Exanthem im Rahmen der durch Parvo-B19-Viren bedingten Pneumonie bei negativer Medikamentenanamnese.

## Therapie

Es erfolgte die Einleitung einer parenteralen Antibiose und einer systemischen Steroidtherapie. Ergänzend erhielt die Patientin eine analgetische und antipyretische Therapie (Paracetamol, Hydal, Neodolpasse). Aufgrund der starken Schwellung im Genitalbereich wurde ein Blasenkatheter gelegt. Ergänzend erfolgten die Gabe von Sauerstoff über die Nasenbrille und parenterale Flüssigkeits- und Elektrolytsubstitution. In der Folge besserte sich der allgemeine und kardiorespiratorische Zustand der Patientin zunehmend.

## Klinische Differenzialdiagnosen

- Akute Kontaktdermatitis
- Intertriginöse Kandidose
- Initialstadium von „staphylococcal scaled skin syndrome“ (SSSS)
- „Symmetric drug-related intertriginous and flexural exanthema“ (SDRIFE)

## Definition und Hintergrund

Das typische Merkmal einer Infektion mit dem humanen Parvovirus B19 bei Kindern ist das Erythema infectiosum. Bei Erwachsenen wurden jedoch verschiedene Hautmanifestationen berichtet: rötelähnliche Erytheme, Henoch-Schönlein-Purpura, papulös-purpuristisches Handschuh- und Sockensyndrom (PPGSS), atypische Erytheme, Exantheme mit Papeln und/oder Pusteln und Erythema multiforme. In der Literatur findet man vereinzelt Fallberichte, bei der eine Infektion mit dem Parvovirus B19 dem charakteristischen Verteilungsmuster des Baboon-Syndroms entspricht [1]. Im Jahr 1984 wurde dieses als Exanthem mit einem sehr charakteristischen Verteilungsmuster, das auf das Gesäß, die intertriginösen Bereiche und die Beugen beschränkt war, beschrieben. Das Reaktionsmuster erhielt den Namen angelehnt an die charakteristische, leuchtend rote Hautfarbe des Gesäßes und Genitalbereiches des Pavians. Damals wurden 3 Fälle nach Gabe von Ampicillin, Nickel und Quecksilber beschrieben, und man ging von einem hämatogenen Kontaktekzem als Reaktion auf diese Medikamente aus [2]. Eine klinisch orientierte Subklassifikation wurde von Miyahara et al. [3] vorgeschlagen, die das Baboon-Syndrom in 4 Gruppen unterteilt: Die ersten 3 umfassen das durch Kontaktallergene ausgelöste Baboon-Syndrom, während die vierte Gruppe dem durch Medikamente ausgelösten Baboon-Syndrom entspricht, das als „symmetric drug-related intertriginous and flexural exanthema“ (SDRIFE) definiert ist [3] und folgende Kriterien aufweist:

1. Exposition gegenüber einem systemisch verabreichten Medikament zum Zeitpunkt der ersten oder wiederholten Gabe (Kontaktallergene ausgeschlossen),
2. scharf abgegrenztes Erythem im Bereich des Gesäßes/Perianalbereiches und/oder V‑förmiges Erythem im Bereich der Leiste/des Perigenitalbereiches,
3. Beteiligung mindestens eines anderen intertriginösen/flexoralen Hautfaltenbereiches,
4. Symmetrie der betroffenen Bereiche und
5. das Fehlen von systemischen Symptomen und Anzeichen [4].

Zusätzlich zu den 4 beschriebenen Gruppen gibt es laut Miyahara et al. [3] eine weitere kleine Gruppe mit der Bezeichnung „infektionsinduzierte Baboon-Syndrom-artige Hautveränderungen“, ein klinisches Bild, wie es sich auch bei der in unserem Fall vorgestellten Patientin mit negativer Medikamentenanamnese und nachgewiesener Parvovirus-B19-Infektion darstellte. Der Begriff Baboon-Syndrom ist heute wohlgemerkt aus ethischen Gründen unzeitgemäß. Historisch wird das Baboon-Syndrom ohnehin mit einem Gesäßerythem gleichgesetzt, das durch die systemische Aufnahme von Quecksilber und anderen Kontaktallergenen verursacht wird. Mangels Begriffalternative wurde der historische Begriff hier aus klinischen Gründen noch benutzt. Zusammenfassend lässt sich festhalten, dass die Infektion mit Parvovirus B19 atypische Hautbefunde auslösen kann einschließlich petechialer und purpuröser Eruptionen, dies zusätzlich zum Erythema infectiosum und dem papulösen purpurösen Handschuh- und Sockensyndrom, die häufiger vorkommen und gut definiert sind [5].

## Fazit für die Praxis

- Bei Patienten mit Exanthem mit Betonung der großen Beugen ist nicht nur an Medikamente als Auslöser zu denken.
- Gerade bei Hautveränderungen, die eine petechiale Komponente aufweisen und dem Verteilungsmuster eines Baboon-Syndroms ähneln, sollte insbesondere auch ein viraler Auslöser – wie in dem vorliegenden Fall Parvovirus B19 – in Betracht gezogen werden.
